# Use of Public Health Promotion Items to Improve Health in Saudi Arabia

**DOI:** 10.5195/cajgh.2013.77

**Published:** 2013-10-03

**Authors:** Khalid Al Aboud, Waleed Jameel, Zaheer Al Asmari, Hamdan Al Osaimy, Awateef Al Sobiani, Samiah Abdul Salam, Yasser Al Zahrani

**Affiliations:** Department of Public Health, King Faisal Hospital, Makkah, Saudi Arabia

**Keywords:** Promotional Gifts, Saudi Arabia, Public Health, e-Health, Disease Prevention

In recent decades, the Saudi Arabian government has prioritized the development of health care services at all levels of care: primary, secondary, and tertiary. This has caused an improvement in health among the Saudi population.^1^,^2^

Despite these improvements, there remains a large problem of language barriers between healthcare providers and their patients.^3^,^4^ Many Saudi health care providers come from other countries and do not speak Arabic. Bridging the gap between providers and their patients is crucial for improving the understanding of disease prevention. In the past, healthcare providers have tried multiple techniques to connect with their patients, including: utilization of the Internet and social media, promotional items like posters and leaflets, and information distributed through text-messaging services.^5^,^6^ Because addressing the language barrier has proven very difficult, experts have sought alternative methods for connecting health care providers to their patients. One potential method is to increase awareness about health care using gifts and promotional items.

The use of gifts with printed public health messages has been suggested as an alternative because it helps to deliver important health care messages to the community.^5^,^7^–^9^ By definition, promotional or advertising gifts are merchandise that promote a company, corporate image, or brand. Some examples of gifts include calendars, mugs, bags, watches, or toys. The messages of these items can be used to promote any area related to public health awareness, such as: dengue fever control, early detection of breast cancer, and immunizations for childhood diseases. These messages must be concise, understandable, and targeted to the proper audience. For example, mugs with information about the importance of blood sugar control may be placed in diabetic clinics.

The use of promotion items has proven successful when used appropriately, as shown among TB patients.^10^ A recent study by the Center for Global Development shows that people were more compliant and enthusiastic when given promotional gifts.

Through use of promotional gifts, health care providers in Saudi Arabia may help change the public perception of health care and medicine, create awareness regarding specific public health messages, as well as improve health care providers’ relationships with their patients, despite language barriers.
